# The implications of globalization on COVID-19 vaccination in Europe

**DOI:** 10.1038/s41598-022-21493-w

**Published:** 2022-10-19

**Authors:** Dan Lupu, Ramona Tiganasu

**Affiliations:** 1 Faculty of Economics and Business Administration, Alexandru Ioan Cuza University of Iasi, Carol I Boulevard, no.22, Iasi, Romania; 2 Faculty of Law, Centre for European Studies, Alexandru Ioan Cuza University of Iasi, Carol I Boulevard, no. 19, Iasi, Romania

**Keywords:** Health care, Medical research

## Abstract

Although globalization has left its mark on economic dynamism, causing conditionalities among various aspects (market openness, production networks, technological and information developments, migratory flows, international cooperation, humanitarian support, etc.), the less pleasant side of it should not be omitted, i.e. the emergence of the framework for the faster diffusion of epidemiological diseases. Thus, with the onset of the SARS-CoV-2 virus, its widespread circulation is a serious challenge for the provision of efficient solutions to combat it, especially in countries with fragile health systems, poor institutional quality and lack of resources. In this paper we aim to investigate the implications of globalization on the COVID-19 vaccination of the population. The period under analysis is January 1, 2021–January 1, 2022, using montly data, and the object of our study are 48 European states. To capture the relationship between globalization and the vaccination rate, we applied regression models, including a number of factors that may influence the progress of vaccination. In order to test the robustness of the results, the two-stage least squares (2SLS) regressions was used. The regression models developed underlined that globalization impacts the degree of vaccination. More globalized economies are more competitive in COVID-19 management, and the significance of this effect comes from better interconnection in global markets and easier access to medical discoveries. At the same time, countries with a higher vaccination rate are associated with higher levels of development. Based on the results obtained, we proposed some policy recommendations to increase the propensity to vaccinate, ensure equity in the distribution of vaccines and provide financial support to developing countries.

## Introduction

The coronavirus outbreak has brought to the attention of authorities and citizens an aspect that is often neglected, that of the appearance of exogenous shocks, which are difficult to control internally^1^. Globalization, despite its many positive implications, can accelerate the emergence of such crises^2^. Theoretical models regarding the effects of globalization on a pandemic showed that a higher degree of integration on international markets can have either positive or negative effects, all depending on the compliance with sanitary recommendations, welfare, company behavior and foreign trade^3–7^.

Globalization is felt on a multidimensional level, involving several decision-makers, which is why proposals and measures to limit the negative impact of the COVID-19 pandemic have not taken long to appear^8^. Thus, one of the studies which examined the role of globalization in adopting restrictions on international flights for 185 countries in 2020 has highlighted that most globalized states have applied restrictive flight policies, with great caution, and only after having a large number of cases of illness^9^. In order to emphasize whether globalization has shaped the spread of COVID-19 in the first wave, it has been concluded that globalization has a stronger effect in countries with more cases, in time this becoming an important influencing factor^10^. Additionally to these findings, a research conducted in 24 countries revealed that a lower degree of globalization had a significant determinism on the spread of the disease in terms of reduced connectivity to the global level, geographical isolation and restrictive measures at the border^11^.

The association of vaccination with globalization has been a challenge over the past decades. Thus, long before the outbreak of the COVID pandemic, it was pointed out that globalization had considerably improved the vaccination rate of the population and the prevention of diseases through a number of mechanisms such as: the harmonization of legislation, the sharing of intellectual property rights through WTO, global fundraising initiatives, vaccine production to a considerable extent for underdeveloped countries^12^. Regarding vaccination policies for adults in developed states, it is shown that for a high percentage of them there is no advanced scheme or health control mechanism^13^, which in the context of the fight against COVID-19 is no longer valid because the desire, on a global scale, is to find the most effective intervention tools, appropriate to internal particularities^14^. Correlating globalization with the impact of the discovery of vaccines, it is stated that when facing a pandemic, humanity takes sustained efforts to accelerate the development of such products^15^. Moreover, by comparing several health crises, it was noticed that the request of the population to be vaccinated against COVID-19, as well as against H1N1 (in 2009), is mainly determined by communications regarding the safety and efficacy of vaccines. In this context, globalization has a particular relevance, manifested through social networks^16^. Clearly, there is a need for stronger international cooperation in the production and distribution of vaccines between countries, pharmaceutical companies, suppliers, health workers and experts, decision-makers and the general population^17^. Although countries located in the South of the world have been less affected by the COVID-19 pandemic^18^, regional cooperation is essential to overcome its economic and health effects, forcing nations to cooperate in the health sector^19^, by aligning national and global policies, strengthening disease surveillance, developing production chains and international trade, encouraging the elaboration of necessary equipment and medicines^20^. There are also explanations for linking population immunization and Sustainable Development Goals for a number of African countries^21^, where the vaccination of the population plays a key role in achieving these goals, although vaccination indicators are low despite ongoing international interventions and aid^22^. At the same time, after the identification of Delta (B1617.2) variant in India and its rapid transmission globally, in the first part of the year 2021, which put strong pressure on health systems, the number of vaccinations was constantly increasing, with a relative reduction in the summer of 2021^23^.

Regardless of the place where these vaccines were developed, the benefits of their widespread application are indisputable^24,25^. Therefore, globalization is an extremely complex and multifaceted socio-economic concept that has a continuous influence on economies, manifesting itself both internally and externally^26^. International institutions such as World Bank and World Health Organization (WHO) consider globalization as a more comprehensive phenomenon (in the detriment of the economic one), which calls for a greater integration of countries and societies beyond geographical borders^27^. In WHO’s view, globalization involves two interconnected phenomena: a flow of factors of production and goods and a series of trade policies, shaped by the fast and wide diffusion of technologies, resulting in a strong interdependence of national economies^28^.

In the last years, the link between globalization in its various aspects (social, economic, political) and health has begun to be the subject of an increasing number of descriptive or empirical studies^29–31^. Such research is the proof of the positive effects that globalization has on health through access to better services, faster transmission of medical information and goods, economic openness, economic integration, liberalization of trade, economic freedom, higher incomes, etc. Increased participation in global commodity circuits and the supply of medical products may lead to high anti-COVID 19 vaccination rates^32,33^. However, depending on the chracteristics of certain countries, some potential mechanisms may appear to work in the opposite direction, with severe negative effects such as migrations of doctors from poor countries, the rapid transmission of infectious diseases, the increase in the number of obese people^34^. With regard to the implications of trade agreements for public health, it was pointed out that they can have mixed effects: increased access to medicines, the consumption of processed foods and carbonated beverages, cardiovascular diseases, life expectancy modeling, a slight growth in infant mortality^8^. Along this line, the impact of global trade on non-communicable diseases (caused by excessive sugar consumption, tobacco, alcohol) was researched and it was outlined that by correlating global policy action, improved results can be achieved^6^. Analyzing the effect of globalization on life expectancy for over 100 countries, in the period 1960–2000, it is emphasized that with the increase of globalization, the gaps in life extension between developed and least developed countries have been reduced^3^, the main cause for this increase being the fast transmission of health equipment and therapies.

In relation to the policies taken by 62 governments in the context of globalization, one study^35^ concludes that measures related to employment and working conditions, alcohol, tobacco and food regulation have the best health outcomes, while the privatization of public utilities and crises have led to decreased outcomes. Starting from the investigation of the effects that trade agreements have on population malnutrition and climate change, another research^36^ highlighted that by liberalizing trade and investment it is possible to improve these problems.

Taking into account all these aspects, the paper aims to study the effects that globalization has on the vaccination of the population. The analysis is performed on 48 European countries and the covered period is January 1, 2021–January 1, 2022, using monthly data. As a measure of globalization we consider the KOF globalization index, proposed by the Swiss Federal Institute of Technology.

The innovative elements of this study are the following: firsly, our research points out the effects of globalization on vaccination (in this regard, currently these is a gap in knowledge); secondly, the research is performed for a consistent sample (48 countries); thirdly, the study proposes a number of implications that can be used by policy-makers and governments. Given that since the end of 2020, thanks to the considerable efforts of health scientists and various institutions, it has been possible to obtain vaccines that have proven to be effective, it is interesting to investigate whether European states manage to reap the benefits of vaccination, generating the premises for the gradual elimination of the COVID-19 pandemic.

After this introductory part, the study continues with a description of the methodological approach and then, the results obtained are presented. The presentation of results is followed by their discussion and conclusions.

## Data and methodology

The central research question is whether globalization influences the COVID-19 vaccination process. To address this challenge, the analysis is structured as follows: identifying variables included in the model, based on the literature; reporting correlations between variables; econometric modeling using OLS regression with robust standard errors; testing the robustness of results using the instrumental variable in a two-stage least squares (2SLS) equation. This approach helps us to examine the determinants of vaccination. One indicator was used as a dependent variable (the percentage of the fully vaccinated population, where fully vaccinated may be single-dose or two-dose, depending on the type of vaccine administered)^37,38^. The sources of the dependent variable were WHO and Worldometer.

The main independent variable is globalization, considered as the composite KOF globalization index, with values between 0 and 100, developed by the Swiss Federal Institute of Technology^26^. This indicator captures various aspects of globalization (economic, cultural, international trade, politics) and a high value implies an advanced degree of integration on global markets. The last year for which the index was available at the time when we carried out our research was 2019.

A number of other possible socio-economic, institutional or health factors may influence the progress of vaccination against COVID-19. Thus, the control variables in our analysis are: GDP per capita^39^, COVID-19 deaths^40^, vaccine certificates^41^, population over 65^42,43^. The data sources are Eurostat, World Bank, WHO, Our World in Data, John Hopins University Coronavirus Resouce Center, and Swiss Federal Institute of Technology. All data were transformed using logarithms.

In order to increase vaccination, several European countries have considered it necessary to implement mandatory green certificates for certain categories or even for the whole population. Thus, these certificates were taken into account as a dummy variable, with values of zero for the countries that have not imposed the certificates and one for the other countries^44^.

Demographic factors are also relevant for vaccination and, in our research, we used the indicator refering to the population over 65 as a percentage of the total population^45–47^. This could influence COVID-19 vaccination because the elderly population, who can also suffer from various diseases, is more likely to become seriously ill. For this reason, it has been included by national governments in the first phase of vaccination.

To study the relationship between COVID-19 vaccination rate and the level of globalization, we apply ordinary least squares (OLS) regression with robust standard errors, seeking to estimate the following model:

1 $$ {\text{Vaccination}}\,{\text{rate}}_{i} \, = \,{\text{Vaccination}}\,{\text{rate}}_{i - 1} \, + \,{\text{Globalization}}_{i} \, \times \,\beta_{1} \, + \,X_{1i} \, \times \,\beta_{2} \, + \,u_{i} , $$

where: i represents the country, vaccination_i_ is the percentage of COVID-19 vaccination of a country; globalization_i_ represents KOF globalization index for country_i_; X_i_ is a country-specific vector that captures different assumptions regarding socio-economic factors (such as the number of deaths, GDP per capita, population over 65, mandatory vaccination).

In our equation, the COVID-19 deaths can shape the intention to vaccinate, and vice versa, the vaccination can reduce the death rate. Under these conditions, COVID-19 death rate can be an instrumental variable in a two-stage least squares (2SLS) equation. Thus, we try to eliminate the potential endogeneity or reverse causality between vaccination and COVID-19 deaths. The instrumental variable depend in our model of COVID-19 cases, GDP per capita, population age. To check the robustness of the obtained results, we apply 2SLS regressions. The testing is performed on the same indicators and models used in OLS regression.

The analysis period for vaccination was January 1, 2021–January 1, 2022, this being based on monthly data. The sample consists of 48 European states (which are found in the appendix), defined by the WHO as belonging to the European continent. The reason for selecting these countries is the similarity of the development of the population’s disease in successive waves and the vaccination campaigns, as well as the relative homogeneity of the adopted public health policies. STATA17 is the program used to perform the empirical analysis.

## Results

Our research is carried out on three levels: firstly, we emphasize the correlations between variables; secondly, there is an OLS regression type analysis including globalization, while thirdly, we highlight the testing of the robustness of the results obtained in the second level using 2SLS, by maintaining the same independent control variables.

Table 1 presents the main descriptive statistics for the variables considered in the study. The average rate of the full vaccination of the population for European countries is 62%, the maximum reached (91%) being registered in Portugal, and the minimum in Bosnia and Herzegovina (24%). The standard deviation is 18.56, which means that there are large variations of the indicator between the considered units. The globalization index has an average value of 80.37 for European countries, with a maximum of 90.79 for Switzerland and a minimum of 66.65 for Albania, the standard deviation being 7.36.

**Table 1:** Descriptive statistics.

	Mean	Max	Min	Std. Dev.
COVID-19 vaccination	62.663	91.867	24.745	18.562
Globalization	80.376	90.793	66.650	7.362
COVID-19 deaths	1878.899	4010.350	99.02100	1002.012
GDP per capita	30,739.64	116,597.3	3726.927	26,705.44
Population over 65	17.712	22.751	8.981	3.095

Table 2 shows the results obtained for correlation analysis. Pearson correlations between vaccination rates and globalization index are statistically significant and positive (0.71). The positive values of these coefficients point out that for the European states, the participation in a more globalized economy is manifested simultaneously with higher COVID-19 vaccination rates. For all indicators, there are positive correlations between them and vaccination rates, stronger for globalization and certificate.

**Table 2:** Correlation test for analyzed variables.

	Vaccination	COVID-19 deaths	Population over 65	Globalization	GDP per capita	Certificates
Vaccination	1	0.589	0.389	0.710	0.650	0.775
COVID-19 deaths	0.589	1	0.104	0.232	0.542	0.418
Population over 65	0.389	0.104	1	0.563	0.074	0.341
Globalization	0.710	0.232	0.563	1	0.618	0.807
GDP per capita	0.650	0.542	0.074	0.618	1	0.749
Certificates	0.775	0.418	0.341	0.807	0.749	1

Table 3 features the main estimates for OLS regression, including the indicators described above. The globalization coefficient is positive and statistically significant for 8 months and models, which highlights that globalization shapes the vaccination of the population. At the beginning of the vaccination period (January–April 2021), globalization did not play an important role in the vaccination process. Subsequently, starting with May 2021, globalization began to have a significant contribution in the vaccination process.

**Table 3:** Relationship between COVID-19 vaccination and globalization (coefficient and probability OLS regressions).

	Jan	Feb	Mar	Apr	May	Jun	Jul	Aug	Sep	Oct	Nov	Dec
Vaccination (− 1)	0.703*** (0.748)	0.824*** (< 0.001)	0.458*** (< 0.001)	0.533*** (< 0.001)	0.640*** (< 0.001)	0.902*** (< 0.001)	0.774*** (< 0.001)	0.857*** (< 0.001)	0.870*** (< 0.001)	0.685*** (< 0.001)	0.852*** (< 0.001)	0.914*** (< 0.001)
Globalization	0.202*** (0.142)	0.225*** (0.248)	0.341*** (0.335)	0.182*** (0.429)	0.089*** (0.021)	0.272*** (0.008)	0.128*** (0.049)	0.106*** (0.002)	0.119*** (0.002)	0.244*** (0.014)	0.172*** (0.011)	0.233*** (0.008)
Certificates	0.282*** (0.630)	0.240*** (0.645)	0.268*** (0.211)	0.182*** (0.312)	0.123*** (0.561)	0.037*** (0.270)	0.310*** (0.025)	0.210*** (0.006)	0.142*** (0.014)	0.390*** (0.005)	0.664*** (0.008)	0.645*** (0.007)
GDP per capita	0.666*** (0.456)	0.474*** (0.528)	0.153*** (0.007)	0.303*** (0.006)	0.292*** (0.407)	0.386*** (0.122)	0.370*** (0.454)	0.410*** (0.010)	0.439*** (0.712)	0.369*** (0.093)	0.385*** (0.244)	0.338*** (0.356)
Population over 65	0.636*** (0.353)	0.095*** (0.822)	0.055*** (0.105)	0.278*** (0.064)	0.130*** (0.381)	0.159*** (0.167)	0.059*** (0.616)	0.055*** (0.490)	0.015*** (0.714)	− 0.069*** (0.758)	0.043*** (0.552)	0.045*** (0.554)
COVID-19 deaths	0.969*** (0.271)	0.764*** (0.026)	0.286*** (0.033)	0.289*** (0.439)	0.259*** (0.516)	0.033*** (0.114)	0.263*** (0.844)	0.174*** (0.371)	0.308*** (0.223)	0.125*** (0.252)	0.329*** (0.865)	0.242*** (0.518)
Constant	− 1.124*** (0.661)	− 1.562*** (0.204)	− 1.174*** (0.704)	− 1.209*** (0.528)	− 1.286*** (0.129)	− 1.234*** (0.735)	− 1.069*** (0.246)	− 1.519*** (0.376)	− 1.340*** (0.101)	− 1.069*** (0.872)	− 1.004*** (0.984)	− 1.543*** (0.118)
Adjusted R-squared	0.493	0.489	0.526	0.592	0.586	0.597	0.579	0.569	0.599	0.562	0.583	0.598
Range of VIF value	1.101	1.621	1.178	1.304	1.366	1.388	1.434	1.325	1.306	1.302	1.305	1.314
	2.827	2.804	2.826	2.871	3.320	3.095	3.695	3.610	3.252	3.317	3.881	3.860

Probabilities are in parentheses.

***Statistical significance at the 1%.

In the initial model, we mantain the socio-economic indicators at a time, in order to see if the effects of globalization are preserved. The influence of globalization is robust with the progress of vaccination; then, GDP per capita and population over 65 were also considered in the analysis. In addition, for all models, the Variance Inflation Factors (VIF) was calculated, to detect multicollinearity, and all VIF are between 1.1 and 3.8, which underlines that multicollinearity is not manifested, the factors being less than 10. Beyond globalization, COVID-19 deaths, certificates and population age are positively associated and statistically significant with vaccination.

Subsequently, we tested the robustness of the outputs obtained. The methodology used is the 2SLS, in which the same independent variables was maintained, in order to observe if the results for vaccination remain significant or not. If these results remain statistically positive and significant, then it can be stated that there are appropriate initial models.

In Table 4, the 2SLS estimates for COVID-19 vaccination are presented. The results for the globalization coefficient are positive and significant for eight models, similarly to results obtained previously, thus validating our analysis. In the case of other indicators (COVID-19 deaths, certificates, population age over 65), positive and significant associations are outlined. The GDP per capita coefficient is also statistically significant and positive, which means that the more developed the countries, the higher the vaccination rate^48^. The indicator referring to the population over 65 has a positive and robust statistical coefficient. The explanation is that most European countries have forced older people, and those most at risk for COVID-19, to get vaccinated. Moreover, it is possible that the dramatic situation, especially in states with a larger aging population (Spain, Italy, etc.), resulting in many deaths, may have led to the collective mentality of the need for large-scale vaccination^49^.

**Table 4:** Robustness check relationship between COVID-19 vaccination and globalization (coefficient and probability two-stage least squares (2SLS) regressions).

	Jan	Feb	Mar	Apr	May	Jun	Jul	Aug	Sep	Oct	Nov	Dec
Vaccination (− 1)	1.317*** (0.587)	0.971*** (0.021)	0.535*** (0.019)	0.594*** (0.036)	0.693*** (0.012)	1.028*** (0.014)	0.937*** (0.002)	0.904*** (0.031)	0.963*** (< 0.001)	0.793*** (0.036)	0.889*** (0.006)	0.965*** (< 0.001)
Globalization	0.278*** (0.747)	0.322*** (0.202)	0.367*** (0.315)	0.225*** (0.430)	0.104*** (0.006)	0.327*** (0.013)	0.154*** (0.001)	0.185*** (0.040)	0.153*** (0.005)	0.365*** (0.033)	0.193*** (0.001)	0.304*** (0.013)
Certificates	0.343*** (0.227)	0.269*** (0.521)	− 0.422*** (0.459)	0.225*** (0.338)	0.291*** (0.225)	0.143*** (0.004)	0.353*** (0.039)	0.306*** (0.034)	0.171*** (0.043)	0.414*** (0.001)	0.747*** (0.005)	0.718*** (0.027)
COVID-19 deaths	0.944*** (0.190)	0.783*** (0.566)	0.293*** (0.599)	0.348*** (0.182)	0.302*** (0.369)	0.044*** (0.115)	0.341*** (0.564)	0.240*** (0.601)	0.326*** (0.261)	0.221*** (0.320)	0.417*** (0.898)	0.331*** (0.867)
Constant	− 1.113*** (0.487)	− 1.537*** (0.843)	− 1.267*** (0.333)	− 1.293*** (0.781)	− 1.380*** (0.875)	− 1.336*** (0.497)	− 1.402*** (0.484)	− 1.978*** (0.576)	− 1.708*** (0.468)	− 1.115*** (0.693)	− 1.117*** (0.885)	− 1.644*** (0.828)
Adjusted R-squared	0.572	0.527	0.569	0.665	0.655	0.673	0.661	0.691	0.689	0.657	0.683	0.646

Probabilities are in parentheses.

***Statistical significance at the 1%.

## Discussions

According to the main research results (Tables 1–4), the relationship between globalization and the vaccination rate is highlighted, this being confirmed by different types of regressions. Table 4 contains estimates of the initial equation, each with three alternative socio-economic indicators, in order to verify robustness. Our analysis shows that countries with a stronger globalization have benefited from high rates of vaccination of the population. Furthermore, countries with greater economic openness and greater integration into international markets may have faster access to COVID-19 vaccine suppliers^12^.

As an important public service, the vaccination of the population is a major challenge for the coordination and engagement capacity of every country^50^. Looking at the results obtained, we can emphasize at least two essential aspects: the impact of globalization on vaccination is indisputable, reflected in the need to ensure an international integration of knowledge, opinions, good practices and a better understanding of this issue^51^; then, the development pattern of each country shapes the vaccination rate—the more developed a country is, the more resources it can allocate for crisis response, extensive vaccination campaigns and vaccine procurement, while in states with systemic vulnerabilities, the ability to withstand and absorb shocks is much lower^52^.

At the same time, in close correlation with globalization, it is clear that the tendency to increase vaccination is found mainly in the member states of the European Union (EU), compared to those that are not part of this structure. This means that institutional coherence, accountability, government effectiveness and transparency in decisions increase the confidence of people in vaccination campaigns and prompt them to accept vaccination more openly^53^. Additionally, the financial support from the EU to the member countries in order to fight against COVID-19 should be taken into account, as well as the relevance of EU involvement in the process of purchasing various vaccines. If in some countries the openness to vaccination was quite high (e.g. Portugal, Denmark, Ireland, Malta), reaching a significant percentage of the population, in others, reluctance prevailed, thus leading to the drastical reduction of the vaccination rate. The acceptability of vaccines is closely dependent on the ability of countries and their specialists to transmit, in a balanced, clear and well informed manner, both the positive and negative effects of vaccination^54^. Moreover, the phenomenon of globalization, which has made possible for these vaccines to be developed so fast, via joint efforts among researchers, doctors, risk experts, etc. is not a neglectable aspect.

If we were to refer to the EU member states, then we would find that the lowest vaccination rates are in the countries where institutional rigidity and government inefficiency coexist, as the obvious consequences of the experiences of the last decades, of social capital, but, at the same time, this highlights the inability to adapt quickly to crisis contexts^55^. These countries, in particular, have a need for the constant involvement of experts in the field of public health, for the existence of a single voice that would raise awareness of the importance of vaccination. In contrast, states with effective governments have been able to implement appropriate public health campaigns^56^.

The study provides evidence that the higher levels of globalization of countries affect the vaccination rate against COVID-19, because they have more resources and are able to invest in health infrastructure and international cooperation. The current crisis should lead to the development of flexible systems to implement appropriate measures for the rapid cooperation between affected countries. A number of trade policies can be improved to strengthen the effective exchange of medical goods and services^57^. At the beginning of the COVID-19 pandemic, certain states resorted to the limitation of the external transfer of drugs, medical equipment, closing their borders and disconnecting themselves from global markets^58^. However, globalization has allowed a rapid exchange of health information between countries and an adequate response to the pandemic. Globalization is a phenomenon related to transport and technology, and the optimal division of labor, being ultimately an irreversible process. The more globalized countries have a more diversified production, being resistant to different types of shocks^59^.

While before COVID-19, health issues were considered local and treated nationally, after the pandemic they were relevant globally and addressed through international collaborations. Medical regulatory agencies (FDA and EMA) have had a common approach to the COVID-19 pandemic, based on an ongoing dialogue. In order to facilitate and accelerate the development and introduction of new vaccines against COVID-19, the establishment of a joint licensing agreement for vaccines is a prerequisite^60^. Health institutions synchronized their main protocols using common information related to the methods, the analysis sample, the effects of COVID-19 vaccine testing. The technological side of globalization has facilitated the multiple collection of data from several vaccine development programs, their use and effectiveness. Through the global treatment of medical problems, health research is supported, identifying and eliminating national bottlenecks generated by specific standards, procedures and schedules. This will lead to an improved access to medical innovation and vaccination^61^.

Globally, the COVID-19 pandemic has generated a focus on innovation in medical research and the widespread manufacture of new drugs, equipment and vaccines. A number of types of vaccines and drugs created with the contribution of biotechnologies, have experienced an extremely rapid development (Pfizer-BioNTech and Moderna mRNA vaccines). The main cause of these fast developments has been extensive research and its shared use globally^62,63^. However, especially with regard to the provision of vaccines to the population and their administration, a number of complications have been felt, generated by supply chains, as well as issues related to acquisition costs, which have been more pronounced among the poorest states. Within them, distribution networks, logistics resources remained obsolete and fragile during the pandemic, leading to reductions and delays in vaccine deliveries, affecting, in fact, the population’s health. Therefore, it is imperative to ensure equity in the distribution of vaccines by providing financial support to developing countries.

## Conclusions

Vaccines are essentially a consequence of globalization, because in order to create them it was necessary to gather the vast knowledge of the medical world from various countries. Looking more closely at the positive effects of globalization, at least one of them can be highlighted: regardless of the country in which a vaccine is produced, it can be administered in any other state. Then, by pooling resources, vaccines were obtained in about a year, and at the beginning of 2022, around 60% of the world’s population received at least one dose, according to Our World in Data^23^. Governments should strive to protect their own citizens, to implement fair and inclusive policies, and in the case of underdeveloped countries, which do not have the possibility to purchase vaccines, international humanitarian aid is required. In addition, vaccination campaigns must be supported by trustworthy people. Typically, countries with a higher vaccination rate are associated with higher levels of development, better education systems, relevant information channels, and government effectiveness. They are directly interested in the benefits of vaccination and also want to keep the borders open. In principle, at the time the pandemic developed, the states that had a strong globalized system were more resilient, the initial and traditional local problems were solved through global approaches and partnerships. Even more, the production and use of COVID-19’s vaccines has been a boost for globalization.

Hesitation and inconsistency in the application of health safety measures can cause variations in vaccination-related behaviors and that is why it is the obligation of the authorities to impose relevant strategies, adapted to the epidemiological situation. With the authorization of various types of vaccines, which have been shown to be effective in clinical trials, states have at their disposal concrete tools to combat the negative effects of the pandemic, beyond the application of health safety measures, such as wearing protective masks or social distancing. Against the background of identifying the Omicron variant in over 89 countries, in December 2021^23^, of a higher rate of contagion than the Delta strain, it becomes clear that at the moment it is the dominant version globally. As a result, there will certainly be a lot of pressure on the health systems, especially on those that have deficiencies in infrastructure, medical staff, communication with citizens and the quality of regulations/policies.

To sum up and draw a general conclusion, it can be stated that the pandemic is far from over, but its shortening is related to the involvement of each of us, to the awareness of its multidimensional effects and to our appropriate action, including here compliance with health safety measures and government directives. Along these elements, COVID-19 vaccination is particularly relevant.

## Data Availability

The data can be extracted from Worldometer, World Health Organization (WHO) and World Bank databases. However, the data used in the analysis will be available upon request.

## Appendix

### The list of countries included in the dataset

Albania, Andorra, Austria, Belarus, Belgium, Bosnia and Herzegovina, Bulgaria, Croatia, Cyprus, Czechia, Denmark, Estonia, Faeroe Islands, Finland, France, Germany, Greece, Hungary, Iceland, Ireland, Italy, Kosovo, Latvia, Liechtenstein, Lithuania, Luxembourg, Malta, Moldova, Monaco, Montenegro, Netherlands, North Macedonia, Norway, Poland, Portugal, Romania, Russia, San Marino, Serbia, Slovakia, Slovenia, Spain, Sweden, Switzerland, Turkey, Ukraine, United Kingdom.

## Abbreviations

SARS-CoV-2: Severe acute respiratory syndrome coronavirus 2
COVID-19: Coronavirus disease 2019
WHO: World health organization
WTO: World trade organization
Max: Maximum
Min: Minimum
GDP: Gross domestic product
FDA: United States of America food and drug administration
EMA: European medicines agency

## References

[1] Van Bavel JJ, Cichocka A, Capraro V (2022). National identity predicts public health support during a global pandemic. Nat. Commun..

[2] Rose A, Walmsley T, Wei D (2021). Spatial transmission of the economic impacts of COVID-19 through international trade. Lett. Spat. Resour. Sci..

[3] Deaton, A. Health in an Age of Globalization*.* National Bureau of Economic Research Working Paper 10669. http://www.nber.org/papers/w10669, 10.3386/w10669 (2004). Accessed 21 Jan 2022.

[4] Barlow P, McKee M, Basu S (2017). The health impact of trade and investment agreements: A quantitative systematic review and network co-citation analysis. Global. Health.

[5] Elmawazini K, Manga P, Nwankwo S (2019). Health gap between developed and developing countries: Does globalization matter?. Econ. Change Restruct..

[6] Cowling K, Thow AM, Pollack Porter K (2018). Analyzing the impacts of global trade and investment on non-communicable diseases and risk factors: A critical review of methodological approaches used in quantitative analyses. Global. Health.

[7] Antràs, P., Redding SJ, Rossi-Hansberg E. Globalization and pandemics, National Bureau of Economic Research. Working Paper 27840. http://www.nber.org/papers/w27840 (2020). Accessed 24 Jan 2022.

[8] Barlow P, van Schalkwyk MC, McKee M, Labonté R, Stuckler D (2021). COVID-19 and the collapse of global trade: Building an effective public health response. Lancet Planet. Health.

[9] Bickley SJ, Chan HF, Skali A (2021). How does globalization affect COVID-19 responses?. Global. Health.

[10] Sigler T, Mahmuda S, Kimpton A (2021). The socio-spatial determinants of COVID-19 diffusion: The impact of globalisation, settlement characteristics and population. Global. Health.

[11] Li Z, Jones C, Ejigu GS (2021). Countries with delayed COVID-19 introduction—Characteristics, drivers, gaps and opportunities. Global. Health.

[12] Milstien JB, Kaddar M, Kieny MP (2006). The impact of globalization on vaccine development and availability. Health Aff..

[13] Bell E, Neri M, Steuten L (2022). Towards a broader assessment of value in vaccines: The BRAVE way forward. Appl. Health Econ. Health Policy.

[14] Kasis A, Timotheou S, Monshizadeh N (2022). Optimal intervention strategies to mitigate the COVID-19 pandemic effects. Sci. Rep..

[15] McGuire K (2021). COVID-19, contagion and vaccine optimism. J. Med. Humanit..

[16] Neumann-Böhme S, Varghese NE, Sabat I (2020). Once we have it, will we use it? A European survey on willingness to be vaccinated against COVID-19. Eur. J. Health Econ..

[17] Golan, M.S., Trump, B.D., Cegan, J.C., Linkov, I. The Vaccine Supply Chain: A Call for Resilience Analytics to Support COVID-19 Vaccine Production and Distribution. In COVID-19: Systemic Risk and Resilience. Risk, Systems and Decisions, (eds Linkov I., Keenan J.M., Trump B.D.) (Springer, 2021) 10.1007/978-3-030-71587-8_22

[18] Arsenault C, Gage A, Kim MK (2022). COVID-19 and resilience of healthcare systems in ten countries. Nat. Med..

[19] Amimo F, Lambert B, Magit A (2021). A review of prospective pathways and impacts of COVID-19 on the accessibility, safety, quality and affordability of essential medicines and vaccines for universal health coverage in Africa. Global. Health.

[20] Karunathilake K (2021). Positive and negative impacts of COVID-19, an analysis with special reference to challenges on the supply chain in South Asian countries. J. Soc. Econ. Dev..

[21] Guzel AE, Arslan U, Acaravci A (2021). The impact of economic, social and political globalization and democracy on life expectancy in low-income countries: Are sustainable development goals contradictory?. Environ. Dev. Sustain..

[22] Decouttere C, De Boeck K, Vandaele N (2021). Advancing sustainable development goals through immunization: A literature review. Global. Health.

[23] Ritchie, H. *et al.* Coronavirus Pandemic (COVID-19), published online at OurWorldInData.org. Retrieved from: https://ourworldindata.org/coronavirus [Online Resource] (2020-2022).

[24] Barrett S (2007). The smallpox eradication game. Public Choice.

[25] Bergh A, Nilsson T (2010). Good for living? On the relationship between globalization and life expectancy. World Devel..

[26] Gygli S, Haelg F, Potrafke N (2019). The KOF globalisation index—Revisited. Rev. Int. Organ..

[27] Dirmesropian S, Wood JG, MacIntyre CR (2016). Economic evaluation of vaccination programmes in older adults and the elderly: Important issues and challenges. Pharmacoeconomics.

[28] Lee K, Grépin KA, Worsnop C (2021). Managing borders during public health emergencies of international concern: A proposed typology of cross-border health measures. Global. Health.

[29] Wu T, Perrings C, Kinzig A (2017). Economic growth, urbanization, globalization and the risks of emerging infectious diseases in China: A review. Ambio.

[30] Gleeson, D., Labonté, R. Methods and Approaches for Measuring the Impact of Trade Agreements on Public Health. In *Trade Agreements and Public Health. Palgrave Studies in Public Health Policy Research*, Palgrave Pivot, Singapore. 10.1007/978-981-15-0485-3_7 (2020).

[31] Schram, A., Townsend, B. International Trade Agreements and Global Health: Pathways and Politics. In Handbook of Global Health, (eds Haring R., Kickbusch I., Ganten D., Moeti M.) (Springer, 2020) 10.1007/978-3-030-05325-3_84-1

[32] Waxman JG, Makov-Assif M, Reis BY (2022). Comparing COVID-19-related hospitalization rates among individuals with infection-induced and vaccine-induced immunity in Israel. Nat. Commun..

[33] Farzanegan MR, Hofmann HP (2021). Effect of public corruption on the COVID-19 immunization progress. Sci. Rep..

[34] Cuevas García-Dorado S, Cornselsen L, Smith R (2019). Economic globalization, nutrition and health: A review of quantitative evidence. Global Health.

[35] Naik Y, Baker P, Ismail SA, Tillmann T, Bash K, Quantz D, Bambra C (2019). Going upstream–an umbrella review of the macroeconomic determinants of health and health inequalities. BMC Public Health.

[36] Friel S, Schram A, Townsend B (2020). The nexus between international trade, food systems, malnutrition and climate change. Nat. Food.

[37] Chantler T, Karafillakis E, Wilson J (2019). Vaccination: Is there a place for penalties for non-compliance?. Appl. Health Econ. Health Policy.

[38] Elhadi M, Alsoufi A, Alhadi A (2021). Knowledge, attitude and acceptance of healthcare workers and the public regarding the COVID-19 vaccine: A cross-sectional study. BMC Public Health.

[39] Wildman J (2021). COVID-19 and income inequality in OECD countries. Eur. J. Health Econ..

[40] Newbold SC, Finnoff D, Thunström L (2020). Effects of physical distancing to control COVID-19 on public health, the economy and the environment. Environ. Resour. Econ..

[41] Oksuz E, Malhan S, Gonen MS, Kutlubay Z, Keskindemirci Y, Tabak F (2021). COVID-19 healthcare cost and length of hospital stay in Turkey: Retrospective analysis from the first peak of the pandemic. Health Econ. Rev..

[42] Bretschger L, Grieg E, Welfens PJJ (2020). COVID-19 infections and fatalities developments: Empirical evidence for OECD countries and newly industrialized economies. Int. Econ. Econ. Policy.

[43] Amdaoud M, Arcuri G, Levratto N (2021). Are regions equal in adversity? A spatial analysis of spread and dynamics of COVID-19 in Europe. Eur. J. Health Econ..

[44] Karnon J (2020). A simple decision analysis of a mandatory lockdown response to the COVID-19 pandemic. Appl. Health Econ. Health Policy.

[45] Ahrenfeldt LJ, Nielsen CR, Möller S (2021). Burden and prevalence of risk factors for severe COVID-19 in the ageing European population—A SHARE-based analysis. J. Public Health.

[46] Wyper GMA, Assunção R, Cuschieri S (2020). Population vulnerability to COVID-19 in Europe: A burden of disease analysis. Arch. Public Health.

[47] Smolić Š, Čipin I, Međimurec P (2021). Access to healthcare for people aged 50+ in Europe during the COVID-19 outbreak. Eur. J. Ageing.

[48] Ceylan RF, Ozkan B, Mulazimogullari E (2020). Historical evidence for economic effects of COVID-19. Eur. J. Health Econ..

[49] Gandjour A (2021). How many intensive care beds are justifiable for hospital pandemic preparedness? A cost-effectiveness analysis for COVID-19 in Germany. Appl. Health Econ. Health Policy.

[50] Alfano V, Ercolano S (2020). The efficacy of lockdown against COVID-19: A cross-country panel analysis. Appl. Health Econ. Health Policy.

[51] Amaya AB, De Lombaerde P (2021). Regional cooperation is essential to combatting health emergencies in the Global South. Global. Health.

[52] Del Llano J, Mestre-Ferrandiz J, Espin J (2021). Public health policies for the common interest: Rethinking EU states’ incentives strategies when a pandemic reshuffles all interests. Eur. J. Health Econ..

[53] Basher SA, Haque AKE (2021). Public policy lessons from the COVID-19 outbreak: How to deal with it in the post-pandemic world?. J. Soc. Econ. Dev..

[54] Palamenghi L, Barello S, Boccia S (2020). Mistrust in biomedical research and vaccine hesitancy: The forefront challenge in the battle against COVID-19 in Italy. Eur. J. Epidemiol..

[55] Arora NK, Pandey P, Egamberdieva D (2021). COVID-19 pandemic: Aggressive research, vaccination, testing and environmental sustainability are the way forward. Environ. Sustain..

[56] Ciravegna L, Michailova S (2021). Why the world economy needs, but will not get, more globalization in the post-COVID-19 decade. J. Int. Bus. Stud..

[57] Qin X, Godil DI, Khan MK (2021). Investigating the effects of COVID-19 and public health expenditure on global supply chain operations: An empirical study. Oper. Manag. Res..

[58] Sarkodie SA, Owusu PA (2021). Global assessment of environment, health and economic impact of the novel coronavirus (COVID-19). Environ. Dev. Sustain..

[59] Schulenburg JMG (2021). COVID-19: Not the time for health economists? A plea for more proactive health economic involvement. Eur. J. Health Econ..

[60] Summan A, Nandi A (2021). Timing of non-pharmaceutical interventions to mitigate COVID-19 transmission and their effects on mobility: A cross-country analysis. Eur. J. Health Econ..

[61] Bíró A, Branyiczki R, Elek P (2021). Time patterns of precautionary health behaviours during an easing phase of the COVID-19 pandemic in Europe. Eur. J. Ageing.

[62] König M, Winkler A (2021). COVID-19: Lockdowns, fatality rates and GDP growth. Intereconomics.

[63] Falcone R, Ansani A, Colì E (2022). Trusting COVID-19 vaccines as individual and social goal. Sci. Rep..

